# The future of fungi: threats and opportunities

**DOI:** 10.1093/g3journal/jkac224

**Published:** 2022-09-30

**Authors:** Nicola T Case, Judith Berman, David S Blehert, Robert A Cramer, Christina Cuomo, Cameron R Currie, Iuliana V Ene, Matthew C Fisher, Lillian K Fritz-Laylin, Aleeza C Gerstein, N Louise Glass, Neil A R Gow, Sarah J Gurr, Chris Todd Hittinger, Tobias M Hohl, Iliyan D Iliev, Timothy Y James, Hailing Jin, Bruce S Klein, James W Kronstad, Jeffrey M Lorch, Victoria McGovern, Aaron P Mitchell, Julia A Segre, Rebecca S Shapiro, Donald C Sheppard, Anita Sil, Jason E Stajich, Eva E Stukenbrock, John W Taylor, Dawn Thompson, Gerard D Wright, Joseph Heitman, Leah E Cowen

**Affiliations:** Department of Molecular Genetics, University of Toronto, Toronto, ON M5G 1M1, Canada; Shmunis School of Biomedical and Cancer Research, The George S. Wise Faculty of Life Sciences, Tel Aviv University, Tel Aviv 69978, Israel; U.S. Geological Survey, National Wildlife Health Center, Madison, WI 53711, USA; Department of Microbiology & Immunology, Geisel School of Medicine at Dartmouth, Hanover, NH 03755, USA; Infectious Disease and Microbiome Program, Broad Institute of MIT and Harvard, Cambridge, MA 02142, USA; Department of Bacteriology, University of Wisconsin-Madison, Madison, WI 53706, USA; Department of Mycology, Institut Pasteur, Université de Paris, Paris 75015, France; MRC Centre for Global Infectious Disease Analysis, Imperial College, London W2 1PG, UK; Department of Biology, University of Massachusetts, Amherst, MA 01003, USA; Department of Microbiology and Department of Statistics, University of Manitoba, Winnipeg, MB R3T 2N2, Canada; Plant and Microbial Biology Department, University of California, Berkeley, CA 94720, USA; Department of Biosciences, University of Exeter, Exeter EX4 4QD, UK; Department of Biosciences, University of Exeter, Exeter EX4 4QD, UK; Laboratory of Genetics, Center for Genomic Science Innovation, J.F. Crow Institute for the Study of Evolution, DOE Great Lakes Bioenergy Research Center, Wisconsin Energy Institute, University of Wisconsin-Madison, Madison, WI 53726, USA; Infectious Disease Service, Department of Medicine, and Immunology Program, Sloan Kettering Institute, New York, NY 10065, USA; Department of Microbiology and Immunology, Weill Cornell Medicine, New York, NY 10065, USA; Department of Ecology and Evolutionary Biology, University of Michigan, Ann Arbor, MI 48109, USA; Department of Microbiology and Plant Pathology, Center for Plant Cell Biology, Institute for Integrative Genome Biology, University of California—Riverside, Riverside, CA 92507, USA; Department of Pediatrics, School of Medicine and Public Health, University of Wisconsin—Madison, Madison, WI 53706, USA; Department of Internal Medicine, School of Medicine and Public Health, University of Wisconsin—Madison, Madison, WI 53706, USA; Department of Medical Microbiology and Immunology, School of Medicine and Public Health, University of Wisconsin—Madison, Madison, WI 53706, USA; Michael Smith Laboratories, University of British Columbia, Vancouver, BC V6T 1Z4, Canada; U.S. Geological Survey, National Wildlife Health Center, Madison, WI 53711, USA; Burroughs Wellcome Fund, Durham, NC 13901, USA; Department of Microbiology, University of Georgia, Athens, GA 30602, USA; Microbial Genomics Section, Translational and Functional Genomics Branch, National Human Genome Research Institute, National Institutes of Health, Bethesda, MD 20892, USA; Department of Molecular and Cellular Biology, University of Guelph, Guelph, ON N1G 2W1, Canada; McGill Interdisciplinary Initiative in Infection and Immunology, Departments of Medicine, Microbiology & Immunology, McGill University, Montreal, QC H3A 0G4, Canada; Department of Microbiology and Immunology, University of California, San Francisco, San Francisco, CA 94117, USA; Department of Microbiology and Plant Pathology, Center for Plant Cell Biology, Institute for Integrative Genome Biology, University of California—Riverside, Riverside, CA 92507, USA; Max Planck Fellow Group Environmental Genomics, Max Planck Institute for Evolutionary Biology, Plön 24306, Germany; Environmental Genomics, Christian-Albrechts University, Kiel 24118, Germany; Department of Plant and Microbial Biology, University of California—Berkeley, Berkeley, CA 94720, USA; LifeMine Therapeutics, Cambridge, MA 02140, USA; M.G. DeGroote Institute for Infectious Disease Research, Department of Biochemistry and Biomedical Sciences, DeGroote School of Medicine, McMaster University, Hamilton, ON L8N 3Z5, Canada; Department of Molecular Genetics and Microbiology, Medicine, and Pharmacology and Cancer Biology, Duke University Medical Center, Durham, NC 27710, USA; Department of Molecular Genetics, University of Toronto, Toronto, ON M5G 1M1, Canada

**Keywords:** ecosystem health, fungal pathogens, medical mycology, plant-pathogenic fungi, sustainability, wildlife pathogens

## Abstract

The fungal kingdom represents an extraordinary diversity of organisms with profound impacts across animal, plant, and ecosystem health. Fungi simultaneously support life, by forming beneficial symbioses with plants and producing life-saving medicines, and bring death, by causing devastating diseases in humans, plants, and animals. With climate change, increased antimicrobial resistance, global trade, environmental degradation, and novel viruses altering the impact of fungi on health and disease, developing new approaches is now more crucial than ever to combat the threats posed by fungi and to harness their extraordinary potential for applications in human health, food supply, and environmental remediation. To address this aim, the Canadian Institute for Advanced Research (CIFAR) and the Burroughs Wellcome Fund convened a workshop to unite leading experts on fungal biology from academia and industry to strategize innovative solutions to global challenges and fungal threats. This report provides recommendations to accelerate fungal research and highlights the major research advances and ideas discussed at the meeting pertaining to 5 major topics: (1) Connections between fungi and climate change and ways to avert climate catastrophe; (2) Fungal threats to humans and ways to mitigate them; (3) Fungal threats to agriculture and food security and approaches to ensure a robust global food supply; (4) Fungal threats to animals and approaches to avoid species collapse and extinction; and (5) Opportunities presented by the fungal kingdom, including novel medicines and enzymes.

##  

**Workshop Participants**

**Alan Bernstein, PhD**, CIFAR President & CEO

**Kate Geddie, PhD**, CIFAR Senior Director, Research

**Louis Muglia, MD, PhD**, Burroughs Wellcome Fund President & CEO

**Victoria McGovern, PhD**, Burroughs Wellcome Fund Senior Program Officer

**Leah Cowen, PhD**, CIFAR Fungal Kingdom Co-Director, University of Toronto

**Joseph Heitman, MD, PhD**, CIFAR Fungal Kingdom Co-Director, Duke University

**Neil Gow, PhD**, CIFAR Fungal Kingdom Advisory Committee Member, University of Exeter

**John Taylor, PhD**, CIFAR Fungal Kingdom Advisory Committee Member, University of California, Berkeley

**David Blehert, PhD**, CIFAR Fungal Kingdom Fellow, U.S. Geological Survey

**Christina Cuomo, PhD**, CIFAR Fungal Kingdom Fellow, Broad Institute

**Cameron Currie, PhD**, CIFAR Fungal Kingdom Fellow, University of Wisconsin-Madison

**Matthew Fisher, PhD**, CIFAR Fungal Kingdom Fellow, Imperial College London

**Lillian Fritz-Laylin, PhD**, CIFAR Fungal Kingdom Fellow, University of Massachusetts Amherst

**Sarah Gurr, PhD**, CIFAR Fungal Kingdom Fellow, University of Exeter

**Timothy James, PhD**, CIFAR Fungal Kingdom Fellow, University of Michigan

**Hailing Jin, PhD**, CIFAR Fungal Kingdom Fellow, University of California, Riverside

**Bruce Klein, MD, PhD**, CIFAR Fungal Kingdom Fellow, University of Wisconsin-Madison

**James Kronstad, PhD**, CIFAR Fungal Kingdom Fellow, University of British Columbia

**Don Sheppard, MD, PhD**, CIFAR Fungal Kingdom Fellow, McGill University

**Jason Stajich, PhD**, CIFAR Fungal Kingdom Fellow, University of California, Riverside

**Eva Stukenbrock, PhD**, CIFAR Fungal Kingdom Fellow, Kiel University and Max Planck Institute of Evolutionary Biology

**Gerard Wright, PhD**, CIFAR Fungal Kingdom Fellow, McMaster University

**Iuliana Ene, PhD**, CIFAR Fungal Kingdom Azrieli Global Scholar, Institut Pasteur

**Aleeza Gerstein, PhD**, CIFAR Fungal Kingdom Azrieli Global Scholar, University of Manitoba

**Rebecca Shapiro, PhD**, CIFAR Fungal Kingdom Azrieli Global Scholar, University of Guelph

**Nicola Case,** CIFAR Fungal Kingdom Meeting Reporter, University of Toronto

**Judith Berman, PhD**, Tel Aviv University

**Robert Cramer, PhD**, Dartmouth

**N. Louise Glass, PhD**, University of California, Berkeley

**Chris Todd Hittinger, PhD**, University of Wisconsin-Madison

**Tobias Hohl, MD, PhD**, Memorial Sloan Kettering Cancer Center

**Iliyan Iliev, PhD**, Weill Cornell Medical College

**Jeffrey Lorch, PhD**, U.S. Geological Survey

**Aaron Mitchell, PhD**, University of Georgia

**Julie Segre, PhD**, National Human Genome Research Institute

**Anita Sil, MD, PhD**, University of California San Francisco

**Dawn Thompson, PhD**, LifeMine Therapeutics, Vice President, Head of Microbiology and Automation

## Introduction

Despite their perception as something found in the forest or served up on a dinner plate, fungi are more than just mushrooms. They span an impressive range of sizes, from microscopic cells to among the largest organisms on Earth (Sipos *et al.* 2018), and have a major impact on human health, agriculture, biodiversity, ecology, manufacturing, and biomedical research. Fungi are key members of aquatic (Grossart *et al.* 2019) and terrestrial ecosystems, both as the Earth's preeminent degraders of organic matter and by forming beneficial symbioses with 90% of land plants (Willis 2018), producing mycorrhizal networks that have come to be known as the “Wood-Wide Web” (Simard *et al.* 1997). Fungal secondary metabolites have revolutionized modern medicine, as exemplified by penicillin, the world's first natural product antibiotic, and immunosuppressive drugs, like cyclosporin that enables organ transplantation, as well as anticancer and cholesterol-lowering drugs (Keller 2019). From bioremediation to biofuels and beer to bread, the applications for which we employ these organisms and their products seem only limited by imagination. For example, there has been an increasing number of patent applications for fungal-based biomaterials with utility in the packaging, textile, leather, housing, and automotive industries (Cerimi *et al.* 2019). While the fungal kingdom clearly presents enormous opportunities, it also poses major threats. Fungi can cause life-threatening bloodstream infections in humans, resulting in at least as many deaths per year as tuberculosis or malaria (Brown, Denning, Gow *et al.* 2012; Brown, Denning, and Levitz 2012). The landscape of these infections continues to change with climate warming, with increases in extreme weather events such as tornados exacerbating human fungal disease (Weinhold 2013). In parallel, fungi are responsible for devastating losses to staple crops that feed billions, jeopardize food security, and cause species declines and extinctions in bat and amphibian species that threaten biodiversity and ecosystem function (Fisher *et al.* 2016, 2020).

Inspired by colloquia held by the American Academy of Microbiology (AAM) in 2007 (Buckley 2007) and 2017 (American Academy of Microbiology 2019) and a long-standing partnership between the Burroughs Wellcome Fund and the mycology community, the Canadian Institute for Advanced Research (CIFAR) held a workshop in November 2021 to help chart the future challenges and opportunities presented by the fungal kingdom. Attended by members of the CIFAR *Fungal Kingdom: Threats & Opportunities* research program (Case *et al.* 2020), the Burroughs Wellcome Fund, and by leading experts on fungal biology from academia and industry, participants convened to strategize on and address questions pertaining to (1) Connections between fungi and climate change and ways to avert climate catastrophe; (2) Fungal threats to humans and ways to mitigate them; (3) Fungal threats to agriculture and food security and approaches to ensure a robust global food supply; (4) Fungal threats to animals and approaches to avoid species collapse and extinction; and (5) Opportunities presented by the fungal kingdom including novel medicines and enzymes. In addition, building on the previous AAM colloquia, participants revisited the recommendations outlined in 2007 (Buckley 2007) and 2017 (American Academy of Microbiology 2019) reports to provide updated suggestions for accelerating fungal research.

## Connections between fungi and climate change and ways to avert climate catastrophe

### How can we alter plant microbiomes to enhance CO_2_ sequestration?

The anthropogenic production of greenhouse gases, such as CO_2_, is expected to raise global temperatures by 2–5°C in the coming decades (Pachauri and Reisinger 2007). Restoring carbon balance by reducing and offsetting emissions is a major environmental sustainability goal (Arora and Mishra 2019) in which vegetation, soils, and oceans play an important role by sequestering carbon, thereby removing it from the atmosphere (Sabine *et al.* 2004; Lal 2005). Mycorrhizal fungi, which form symbioses with plant roots, have a remarkable impact on soil carbon sequestration (Adamczyk 2021), wherein forest ecosystems dominated by different types of mycorrhizal fungi have vastly different carbon storage capabilities (Averill *et al.* 2014). Building on this knowledge, participants suggested enhanced research in precision forest mycobiome engineering as a strategy to augment carbon sequestration by forests. In addition to contributing to carbon sequestration (Clemmensen *et al.* 2013), fungi respire CO_2_ and can cause considerable soil carbon losses (Cheng *et al.* 2012), as well as release CO_2_ from dead organic matter by contributing to its decomposition. Thus, as highlighted recently (Tiedje *et al.* 2022), the impact of mycobiome engineering on both greenhouse gas sequestration and release would need to be assessed.

Reforestation after clear-cutting for timber harvest, tree planting to offset carbon emission, and crop farming were highlighted as existing practices where engineered mycobiomes could be applied to enhance carbon sequestration. In addition, the group suggested further investigation of the mycobiomes of seaweeds (Suryanarayanan 2012), whose aquatic farming is the fastest-growing component of global food production (Duarte *et al.* 2017). Seaweeds function as important marine CO_2_ sinks (Krause-Jensen and Duarte 2016) and thus understanding how fungi impact the ability of seaweed to fix CO_2_, and whether seaweed-associated fungi can be manipulated to enhance this process, are important to address. Despite the potential of these strategies to enhance CO_2_ sequestration, participants identified several challenges. The importance of understanding local ecology to inform decisions on the type and combination of fungi was emphasized, as each environment has its own distinct set of biotic and abiotic factors that are likely to affect the longevity and function of the applied fungal community. Furthermore, the impact of introducing designer fungal communities on native microbes, and other potential ecological consequences, would need to be carefully considered.

### How can we identify new fungal pathogens of crops and existing fungal pathogens whose geographic range is expanding due to climate change?

While fungal symbionts of plants hold promise to enhance carbon capture, fungal plant pathogens are responsible for staggering reductions in absorbed CO_2_ by causing disease (Fisher *et al.* 2012). Even more concerning is evidence that fungal pathogens of crops are moving poleward as the global climate warms (Bebber *et al.* 2013), facilitating the interactions of pathogens with naïve hosts and environments (Chaloner *et al.* 2021). Participants discussed increased surveillance efforts to detect and monitor fungal pathogens of crops as a proactive strategy to improve responsiveness to emerging threats. Efforts would ideally take the form of a global monitoring program, wherein widespread metagenomic sequencing of fungal communities could facilitate the identification of novel pathogens, as well as the movement of known pathogens into new areas. Recognizing the scale of such a program, the group suggested that engaging farmers in citizen science initiatives to aid with sample collection or capturing the interest of industry partners would be beneficial. In all cases, promoting open science and sharing of crop pathogen surveillance and sequencing data as they become available, such as through OpenRiceBlast and OpenWheatBlast (Kamoun *et al.* 2019), would be empowering. In concert with increased local monitoring for crop pathogens, participants emphasized the benefits of enhanced surveillance of internationally transported plants, given the ability of global trade to exacerbate the spread of fungal pathogens (Fisher *et al.* 2020).

### How do fungi respond and adapt to climate change and increasing temperatures?

In addition to expanding the habitat of fungal pathogens, climate warming has the potential to select for environmental fungi adapted for growth at temperatures approaching that of the human body (Garcia-Solache and Casadevall 2010). This poses a major problem because mammalian body temperature acts as a restrictive barrier to fungal infection given that most fungi thrive within the range of 12–30°C (Robert and Casadevall 2009). Thus, if environmental fungi that are currently unable to cause infections in humans evolve increased temperature tolerance, many additional species may become pathogenic (Garcia-Solache and Casadevall 2010). Previous work drawing on archived culture collection data identified fungal genera with a disproportionate number of thermotolerant species, highlighting these genera as potential sources of emerging pathogenic fungi given their propensity for adaptation to higher temperature growth (Robert and Casadevall 2009). Although this study offers a starting point, participants suggested that a large survey of fungal temperature tolerance could be conducted to identify species with the greatest likelihood of overcoming the mammalian thermal barrier. In tandem, the characterization of fungal species that are close relatives to known pathogens, but currently lack thermotolerance, would be beneficial (Garcia-Solache and Casadevall 2010). Such surveys are of increasing importance given the recent report that human body temperatures have decreased over the past century (Protsiv *et al.* 2020), further narrowing the thermal barrier.

## Fungal threats to humans and ways to mitigate these threats

### How did *Candida auris* emerge to cause disease globally and where is it present in the environment?

*Candida auris* is hypothesized to be the first human fungal pathogen to emerge due to thermal adaptation in response to climate change (Casadevall *et al.* 2019); however, its origin is still a mystery. Initially detected in 2009 (Satoh *et al.* 2009), *C. auris* emerged near-simultaneously on 3 continents (Lockhart *et al.* 2017) and has since spread across the globe (Chakrabarti and Sood 2021). *C.auris* poses a major new threat to human health due to its high rate of antifungal resistance, ability to persist on hospital surfaces, and rising number of cases (Chakrabarti and Sood 2021). Recently, *C. auris* was isolated environmentally from a salt marsh and sandy beach on the Andaman Islands in India, suggesting it may be associated with the marine ecosystem (Arora *et al.* 2021). Some participants postulated that prior to its emergence as a human fungal pathogen, *C. auris* may have transiently colonized human skin several times before being carried into a hospital environment and exposed to a susceptible host, leading to amplification and outbreaks. In partial alignment with this hypothesis, screening for *C. auris* skin colonization of patients in a nursing facility identified multiple skin sites including nares, fingers, and toe webs as frequently colonized body sites (Proctor *et al.* 2021). The group suggested that additional environmental sampling for *C. auris* in conjunction with studies of the human skin mycobiome would be beneficial, especially in areas where *C. auris* is endemic.

### Are agricultural practices driving antifungal drug resistance in species beyond *Aspergillus*?

Azoles are widely deployed in agriculture as fungicides but are also used as therapeutics to treat fungal infections in humans and animals (Fisher *et al.* 2018). The dual use of azoles in agriculture and in the clinic has led to the global emergence of azole resistance in the major human fungal pathogen *Aspergillus fumigatus* (Meis et al. 2016; Fisher *et al.* 2018). As a result, azoles are losing utility as a frontline antifungal therapy, leading to increases in patient mortality (Meis et al. 2016). Azole resistance is also exceedingly common in isolates of *C. auris* (Chow *et al.* 2020), raising the question of whether the extensive use of azoles in agriculture also promoted the development of resistance in this emergent fungal pathogen. In addition, participants highlighted that consumption of azole-treated foods may impact resistance in commensal fungi, including those with the potential to be pathogenic. The identification of azole resistance in multiple fungal pathogens underscores the timeliness for application of a ‘One Health’ perspective to antifungal drug deployment, which recognizes that human, plant, and animal health are interconnected (American Academy of Microbiology 2019; Fisher and Murray 2021). Applying a ‘One Health’ approach recognizes stewardship of existing compounds as well as new methods for treating and preventing fungal infections in plants and humans (Fisher *et al.* 2018; American Academy of Microbiology 2019). Strategies for curbing resistance to antifungals could include retaining newly developed therapies for use in either agriculture or medicine. However, limiting antifungals with broad utility for clinical use alone would likely require the implementation of government regulations or incentives given the larger market for use in agriculture.

### How can we accelerate fungal vaccine development and promote the development of newer antifungals and their approval?

New strategies are needed to combat human fungal pathogens given the rising resistance to currently available antifungals and ever-changing landscape of human disease, with novel viruses like SARS-CoV2 producing new patient populations that are vulnerable to fungal infections (Gangneux *et al.* 2022; Hoenigl *et al.* 2022). Despite the substantial health burden posed by fungal pathogens and numerous efficacious vaccines against bacteria and viruses, there are no clinically approved vaccines or monoclonal antibodies to protect against fungal infections (Oliveira *et al.* 2021). Although several fungal vaccines (Oliveira *et al.* 2021), immunotherapies (Da Glória Sousa *et al.* 2011), and monoclonal antibodies (Rudkin *et al.* 2018) are in development and some have reached clinical trials, challenges pose barriers to the fungal vaccine pipeline, recently reviewed in Oliveira *et al.* (2021). Gut commensal fungi can induce germinal center B-cell expansion and systemic antibodies that are protective against disseminated *Candida albicans* or *C. auris* infections, highlighting new opportunities for exploration of the natural human antibody repertoire against gut mycobiota for development purposes (Doron *et al.* 2021). Major advances in mRNA vaccine technology, largely fueled by the ongoing coronavirus disease 2019 (COVID-19) pandemic (Chaudhary *et al.* 2021), present an opportunity for the development of novel fungal vaccines. In all cases, it would be prudent to explore the biological and immunological implications of fungal vaccines on commensal fungi, fungi consumed as food, and fungal allergens, which are currently unknown. In the development of fungal vaccines, immunotherapies, and antifungals, the group highlighted major regulatory, licensing, and distribution barriers, as well as a lack of financial incentives, as roadblocks. Many of the same problems have been identified in the antibiotics pipeline, which led the United Kingdom to trial a model wherein antibiotics are paid for through a subscription, rather than on a per-pill basis (Glover *et al.* 2019; Mahase 2020). A similar pilot program, the Pioneering Antibiotic Subscriptions to End Upsurging Resistance (PASTEUR) Act, was introduced to the United States Congress in 2021 (Outterson 2021). Some participants thought the antifungal pipeline could also benefit from governments buying into a “subscription model” for antifungals, which would decouple revenue from the volume of drugs sold to help encourage new development by offsetting costs.

## Fungal threats to agriculture and food security and approaches to ensure a robust global food supply

### Are there ways to breed crops for resistance or new fungicides to deploy such as those based on RNA?

Genetically modifying crops to enhance resistance to microbial infection offers an alternative to chemical agents (Dong and Ronald 2019). However, genetically modified organisms (GMOs) are banned in a number of countries, including many in the European Union, necessitating alternative approaches for crop protection (Turnbull *et al.* 2021). A promising avenue is the use of RNA interference, a cellular process whereby gene transcript expression is reduced in a sequence-specific manner without modifying the genome (Hernández-Soto and Chacón-Cerdas 2021), thereby circumventing the regulatory processes that limit GMOs. In a method known as spray-induced gene silencing (SIGS), RNAs targeting pathways essential for growth or virulence of the pathogen are sprayed on the plant (Hernández-Soto and Chacón-Cerdas 2021). These RNAs are subsequently taken up by the pathogen, where they act inside the cell to inhibit growth or virulence, thereby protecting the plant from infection (Hernández-Soto and Chacón-Cerdas 2021). Although SIGS may offer a versatile, effective, safe, and eco-friendly approach for crop protection, the group highlighted the need to explore potential off-target effects of SIGS on fungal symbionts of plants, like endophytes and mycorrhizae. Research on the evolution of resistance to RNA uptake was also emphasized as an important area for future research, given that the effectiveness of SIGS for fungal disease control is dependent on the efficiency of RNA uptake by the pathogen (Qiao *et al.* 2021). In addition, some participants highlighted mycoviruses, which are viruses that infect fungi, as potential biological control agents for crop fungal disease (Nuss 2005; Xie and Jiang 2014). Although mycoviruses have been identified to cause reduced virulence in some fungal crop pathogens (Cho *et al.* 2013; Pearson and Bailey 2013), relatively little is known about mycoviruses, underscoring that additional sampling and sequencing to detect mycoviruses, as well as assays to determine their phenotypic effects on different fungal species would be valuable.

### Can we modify the plant mycobiome to enhance resistance to pathogens?

Fungal endophytes, which live within plant tissues, have been widely reported to protect their host plants against herbivore pests (Bamisile *et al.* 2018). Less well studied is the ability of fungal endophytes to protect plants against fungal plant pathogens, although some cases have been reported (Bamisile *et al.* 2018). Despite the promise of fungal endophytes to act as biological control agents against plant fungal pathogens, the group identified 3 major challenges to overcome. First, more insight into the mechanisms by which fungal endophytes confer protection, whether it be directly, through microbial competition or secretion of compounds with antifungal activity, or indirectly, through priming the host immune system, would be beneficial. Second, the best method and location (roots, stem, leaves, or soil) for applying beneficial symbionts would need further investigation. Last, a plant-, pathogen-, and environment-specific approach would be valuable to identify endophyte combinations that lead to stable colonization and effective, long-term protection. Thus, a “look locally first” strategy could be applied, where beneficial endophytes are identified within the local environment, then reapplied in combinations to the host plant. Such a strategy would limit the transport of fungi into non-native ecosystems and ensure endophyte cocktails are adapted to the environment in which they are applied, promoting longevity.

### How do we protect crops from postharvest fungal damage?

While microbial pathogens cause approximately 15% losses in yield by damage to crops in the field (Oerke 2006), the destruction caused by postharvest disease amounts to an additional 20–25% reduction, depending on the country (Sharma *et al.* 2009). Current postharvest disease mitigation strategies rely heavily on chemicals, which pose a threat to human health and the environment. Similar to its utility in protecting preharvest crops, SIGS has been identified as a method for safe and powerful plant protection of postharvest products (Wang *et al.* 2017). In addition, plant (Utama *et al.* 2002) and bacterial (Gao *et al.* 2018; Carter-House *et al.* 2020) volatiles are being investigated for their ability to suppress the growth of fungal pathogens that commonly cause postharvest decay. Some members of the group proposed that microbes that produce fungal growth-inhibiting volatiles could be applied to the packaging of postharvest products to provide a continuous source of volatiles to suppress fungal growth. Padding is already routinely included in the packaging of many fruits to absorb moisture and provide cushioning to prevent damage, thus microbes could readily be applied to these pads to provide a natural solution to postharvest decay caused by fungi. However, care would be needed to ensure the applied microbes cannot cause decay themselves or readily adapt to do so and do not pose a threat to human health.

## Fungal threats to animals and approaches to avoid species collapse and extinction

### Are there ways to protect frogs and salamanders from chytrid pathogens?

The amphibian fungal disease chytridiomycosis, caused by *Batrachochytrium dendrobatidis* and *Batrachochytrium salamandrivorans*, has driven global declines and extinctions in over 500 species of amphibians, amounting to the greatest recorded loss of biodiversity attributable to a pathogen (Scheele *et al.* 2019). International trade in amphibians has been linked to the spread of chytrid pathogens and their introduction into naïve hosts, resulting in disease outbreaks (O’Hanlon *et al.* 2018). Strengthening of transcontinental biosecurity, such as restrictions on salamander import, have helped mitigate disease transmission (Klocke *et al.* 2017), but continued precautions and strategies for supporting the recovery of endangered populations are needed. Participants emphasized that domestic amphibian breeding programs could be implemented to provide a supply of disease-free animals to the pet trade. Domestic breeding programs would prevent large-scale mining of amphibians from the wild, thereby enabling more stringent import regulations to limit the continued global spread of chytrid pathogens.

Strategies for mitigating the impact of chytridiomycosis on wild amphibian populations would also be valuable. Diverse avenues have been explored, such as antifungal treatment of tadpoles coupled with environmental disinfection (Bosch *et al.* 2015), and probiotic therapy through bioaugmentation of microbes that confer defense against chytrids (Bletz *et al.* 2013; Kearns *et al.* 2017; Woodhams *et al.* 2020); but the broad applicability and scalability of these methods have yet to be determined. Additional strategies discussed by the group included biological control using mycoviruses, which has been applied against the fungal agent responsible for chestnut blight (Rigling and Prospero 2018), and the introduction of nonpathogenic chytrid lineages to outcompete virulent lineages. However, more research into the potential impacts of introducing novel mycoviruses or less pathogenic chytrid strains warrant careful study before implementation.

### How can we reduce the impact of bat white-nose syndrome on bat populations and ecosystems?

Bats play a crucial role in maintaining ecosystem health as seed dispersers, pollinators, and controllers of insect pests (Ramírez-Fráncel *et al.* 2022). White-nose syndrome (WNS) is a devastating disease affecting North American hibernating bat populations that is caused by the fungus *Pseudogymnoascus destructans* (Lorch *et al.* 2011). Introduced to North America by humans in 2006 (Blehert *et al.* 2009), *P. destructans* is spreading across the continent, resulting in declines of more than 90% in some bat populations (Turner *et al.* 2011). One strategy for controlling WNS involves vaccinating bats with *P. destructans* antigens to elicit a protective immune response (Rocke *et al.* 2019). Vaccine administration was successful at reducing *P. destructans* infection of bats in a laboratory trial (Rocke *et al.* 2019) and is currently being explored in field trials; however, additional funding would be needed to enable vaccination of afflicted populations. The group suggested raising public awareness of WNS through events such as Bat Week, and attracting companies interested in aging research, given the exceptional longevity of bats (Brunet-Rossinni and Austad 2004), as strategies for garnering financial support. Raising public awareness would also be important to mitigate the spread of WNS by people exploring caves where bats live. The additional methods for managing WNS were discussed, including the use of immune receptor agonists to boost the bat antifungal response to *P. destructans* and sterilization or modification of bat hibernacula sediments, which are a known reservoir of *P. destructans* (Verant *et al.* 2018), to remove the pathogen or suppress its growth with antagonistic microbes.

### How can we increase awareness of other fungal pathogens of animals and the dangers they pose?

In addition to causing devastating disease in amphibians and bats, emerging fungal diseases have been identified in wild snakes (Lorch *et al.* 2015), sea turtles (Sarmiento-Ramírez *et al.* 2014), lizards (Peterson *et al.* 2020), dolphins and porpoises (Teman *et al.* 2021), and birds (Arné *et al.* 2021). Despite this, fungi are often overlooked as sources of emerging infectious disease, compounding their threat to wildlife and ecosystem health (Fisher *et al.* 2020). Given the impact of human movement on spreading infectious diseases of wildlife, participants suggested that developing communication strategies to increase public awareness of the threats fungi pose to wildlife, for use in venues such as schools and national parks, would be beneficial. Public involvement through citizen science and other nationally coordinated programs to report dead wildlife was also proposed as a strategy, which could aid in identifying trends that may indicate the emergence of infectious disease in wildlife populations. As wildlife diseases are inherently difficult to treat, the group highlighted the possible need for a paradigm shift in wildlife disease management from reactive to proactive strategies. A proactive strategy could center on managing wildlife populations for health, which would entail identifying underlying drivers of wildlife infectious diseases and mitigating these factors to prevent disease emergence. It would also involve supporting ecosystem health to help wildlife populations build resilience in the face of disease and enable recovery. Such a strategy may prove effective given the potential for wildlife populations to evolve resistance and recover from disease without human intervention, as has been predicted in bat populations affected by WNS (Auteri and Knowles 2020) and documented in amphibian populations afflicted with chytridiomycosis (Scheele *et al.* 2014).

## Opportunities presented by the fungal kingdom including novel medicines and enzymes

### How can we accelerate discovery of the biological activities of fungal natural products?

Fungi produce an astounding array of natural products, some of which have been developed into drugs that have revolutionized patient care (Keller 2019). The fungal kingdom is exceptionally diverse, home to an estimated 2.2–3.8 million species, the majority of which have yet to be identified (Hawksworth and Lücking 2017). However, progress has been slow in identifying new fungal metabolites that can be advanced into the clinic, partly owing to rediscovery of known molecules. The group discussed using CRISPR genome engineering to minimize rediscovery by inactivating biosynthetic gene clusters (BGCs) needed to make commonly identified antimicrobials in strains of interest (Culp *et al.* 2019) and posited that this approach could be applied on a large scale to existing strain collections or environmental samples to augment the discovery of new natural products. Investigating the molecules produced by animal-associated microbes was also proposed as a strategy to accelerate the pace of drug discovery, given that these microbes appear to be enriched in compounds with low toxicity to animals (Chevrette *et al.* 2019). In addition, genomics was highlighted as an invaluable tool for fungal natural product discovery as genes involved in the biosynthesis of fungal secondary metabolites are often arranged in BGCs, which can be predicted by algorithms (Keller 2019). Thus, the sequencing of fungal genomes can provide insight into the molecules that fungi have the potential to produce, and when coupled with synthetic biology can enable BGCs from unculturable fungi to be expressed in heterologous microbial hosts (Clevenger *et al.* 2017). Lastly, participants underscored the benefit of supporting and sustaining fungal culture collections and databases given their value in enabling natural product discovery and fungal research more broadly.

### Can we harness fungi to develop clean fuels that help avert climate catastrophe?

Fungi have phenomenal potential for applications in bioremediation (Harms *et al.* 2011; Kumar and Chandra 2020) and in the production of sustainable energy sources, such as in the biofuel industry and advanced biorefineries (Hong and Nielsen 2012; Srivastava *et al.* 2018; Keasling *et al.* 2021). Fungi and fungal enzymes are being explored for their ability to convert renewable lignocellulosic materials (plant dry matter) into biofuels, providing environmentally friendly and sustainable alternatives to fossil-derived fuels and chemicals (Srivastava *et al.* 2018; Lillington *et al.* 2021). Moreover, fungi possess the biochemical and ecological capacity to degrade environmental pollutants, such as toxic chemicals generated during textile and pulp production, pesticides, pharmaceuticals, plastics, and crude oil (Harms *et al.* 2011; Zeghal *et al.* 2021). Thus, fungi harbor key enzymes and are key bioconversion chassis both for use in fuel production and its cleanup. The group emphasized the importance of industry-academia partnerships to enable the exploitation of fungi for biofuel production and bioremediation, as well as in general to make broad utilization of fungal enzymes in biotechnology and fungal natural products in drug discovery. Consulting and participation in scientific advisory boards were highlighted as avenues for building industry-academia collaborations, which can promote sharing of expertise and resources to accelerate innovation and application of fungal-derived products. Moreover, participants discussed the value of an increase in initiatives to promote these partnerships, such as programs offering funding to graduate students and postdoctoral fellows, to enable joint industrial-academic research projects.

## Recommendations

**Fig. 1.**
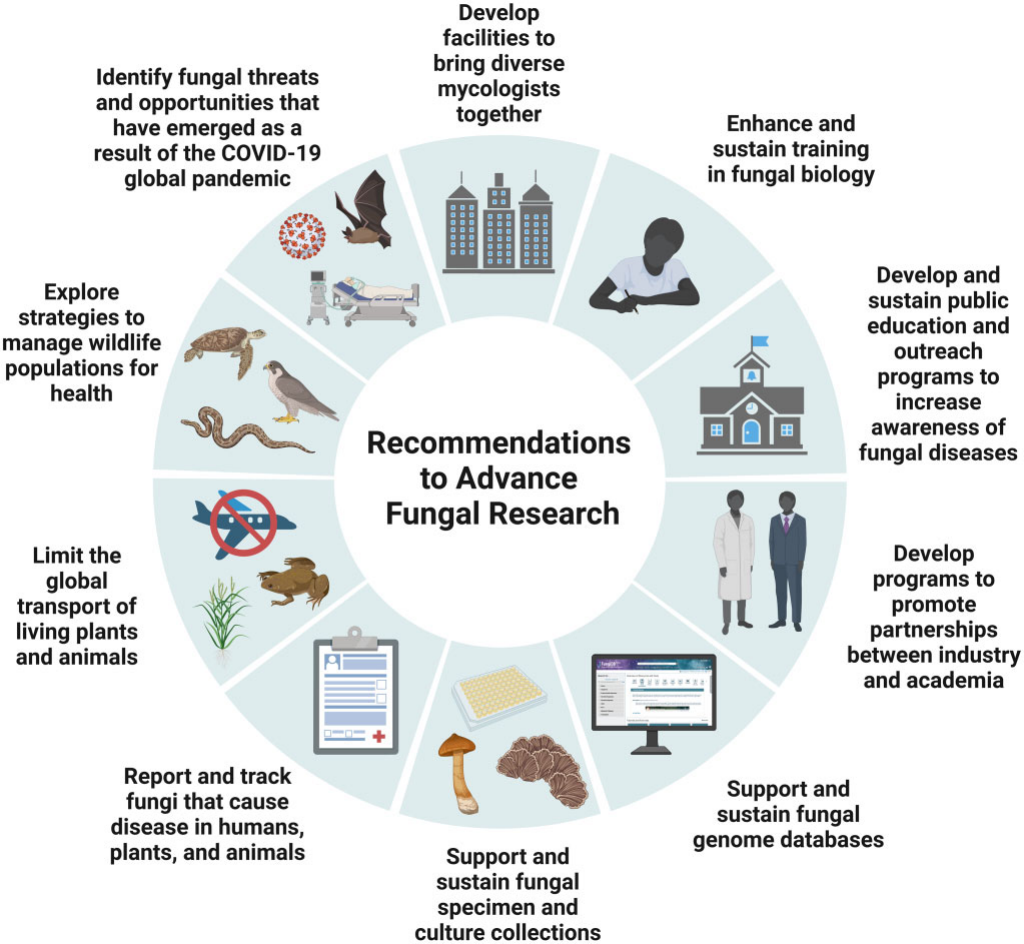
Recommendations to promote advances in fungal research. Created with BioRender.com.

The 2007 (Buckley 2007) and 2017 (American Academy of Microbiology 2019) AAM colloquia reports on the fungal kingdom provided recommendations to promote advances in fungal research. These recommendations were revisited during the meeting hosted by CIFAR and the Burroughs Wellcome Fund and updated to reflect the current state of the world’s fungi and to provide suggestions for accelerating the development of novel strategies to combat the threats posed by fungi and harness their extraordinary potential (Fig. 1). These recommendations have been proposed within the context of relevant background information in the main text and are further distilled in a succinct format below.

1. *Develop Facilities to Bring Diverse Mycologists Together*. An interdisciplinary approach is crucial to mitigating fungal threats and identifying fungal-based solutions to global challenges. Mycology Centers of Excellence would be valuable to coordinate multidisciplinary work between mycologists that focus on fungi that infect humans, plants, and wildlife. Mycology Centers could be modeled after National Institutes of Health (NIH) Cancer Centers and would enable efficient growth and translation of scientific knowledge for application in medicine, agriculture, and ecosystem conservation.
2. *Enhance and Sustain Training in Fungal Biology.* There is a clear benefit to training more individuals to address the diverse facets of fungal biology and the impact of fungi on human, plant, and animal health. In particular, enhanced training in medical mycology, especially in areas where mycoses are endemic, would improve accurate diagnosis and informed treatment of patients. Moreover, the development of programs to train domestic animal and wildlife veterinarians, and to include them in discussions on managing fungal diseases of livestock and wild animals, would serve to further mitigate the impacts of fungi on animal health.
3. *Develop and Sustain Public Education and Outreach Programs to Increase Awareness of Fungal Diseases.* Fungi are not widely regarded as major agents of infectious disease, despite the substantial burden they pose to human, plant, and animal health. Programs could be developed for implementation in schools and national parks to increase public awareness of the threats posed by fungal pathogens and the actions by which humans contribute to their spread.
4. *Develop Programs to Promote Partnerships Between Industry and Academia.* Programs offering financial support, such as funding of graduate students and postdoctoral fellows, would promote collaboration between industry and academia. In addition, these partnerships would enable sharing of resources and expertise, as well as recruit talent to work in industry, ultimately accelerating the pace of innovation.
5. *Support and Sustain Fungal Genome Databases.* Fungal genomics, transcriptomics, proteomics, and other “omics” methods, as well as an increasing number of fungal genome sequences, have generated abundant and rich datasets. Compiling, storing, and annotating these data in an integrated format that is accessible to the community is critical to informing research and promoting advances. Developing and implementing unified taxonomy and standardized pipelines for next-generation sequencing methods would be beneficial.
6. *Support and Sustain Fungal Specimen and Culture Collections.* Fungal specimen and culture collections are critical resources for understanding fungal biology, genetic variation, and evolution. These collections are likely to become increasingly important for examining the impact of climate change on fungi, tracking invasive species, and preserving fungal diversity. Moreover, fungal culture collections represent a rich source of chemical diversity for the identification of natural products that can be developed for clinical use. The long-term availability of these existing and new collections would be valuable in enabling fungal research.
7. *Report and Track Fungi That Cause Disease in Humans, Plants, and Animals.* Documenting the global burden of fungal disease would enable increased understanding of disease emergence, range expansion, and the impact of drug resistance. To achieve this objective, public, domestic animal, and wildlife health agencies could implement programs to transparently report cases of fungal disease, and crop monitoring services could report fungal disease surveillance data to publicly accessible databases.
8. *Limit the Global Transport of Living Plants and Animals.* Global trade can promote the spread of plant and animal fungal pathogens (Fisher *et al.* 2020). Enhanced pathogen surveillance of internationally transported plants and animals coupled with stringent import regulations could limit the introduction of potential pathogens to naïve populations. Locally maintained, disease-free nursery and animal stocks would reduce the need for global import and help prevent the spread of fungal pathogens.
9. *Explore Strategies to Manage Wildlife Populations for Health.* Management of wildlife infectious diseases has historically been reactive despite these diseases being difficult to treat. A paradigm shift toward proactive strategies that act to or are predicted to support ecosystem health and enable wildlife populations to build resilience and recover in the face of disease would be beneficial.
10. *Identify Fungal Threats and Opportunities That Have Emerged as a Result of the COVID-19 Global Pandemic.* The COVID-19 pandemic has produced new patient populations that are vulnerable to fungal infection (Baddley *et al.* 2021). Further research would be important to understand COVID-19-associated fungal diseases, such as mucormycosis (Baddley *et al.* 2021). Concurrently, a Global Virome Project has been launched to discover zoonotic viral threats and stop future pandemics, which will entail sampling of bats and other animals to identify viruses (Carroll *et al.* 2018). This initiative presents both an opportunity, to sample wildlife for fungi in tandem with viruses, and a threat, as human movement during sampling has the potential to spread disease between wildlife populations.

## Conclusions and outlook

The fungal kingdom presents enormous opportunities for applications in medicine, biotechnology, and environmental sustainability, while also posing devastating threats to human, plant, and animal health. Moreover, the breadth of fungal diversity remains relatively underexplored and the impact of climate change and emergent infectious diseases like COVID-19 on fungi have yet to be appreciated. Novel approaches would be beneficial to harness fungi to avert climate catastrophe, support plant health, mitigate wildlife disease, and identify new medicines. The goal of this report is to raise awareness on the diverse ways fungi impact health and disease, to spark innovative solutions to global challenges and fungal threats, and to provide suggestions for advancing the field of fungal biology. As highlighted in the recommendations herein, developing and sustaining infrastructure to support fungal research would catalyze the development of new strategies to mitigate the threats posed by fungi and harness their extraordinary potential.

## Data Availability

There are no new data associated with this article.
